# Case Report: The success of face analysis technology in extremely rare genetic diseases in Korea: Tatton–Brown–Rahman syndrome and Say–Barber –Biesecker–Young–Simpson variant of ohdo syndrome

**DOI:** 10.3389/fgene.2022.903199

**Published:** 2022-08-03

**Authors:** Sunha Park, Jaewon Kim, Tae-Young Song, Dae-Hyun Jang

**Affiliations:** Department of Rehabilitation Medicine, Incheon St. Mary’s Hospital, College of Medicine, The Catholic University of Korea, Seoul, South Korea

**Keywords:** rare diseases, tatton-Brown-rahman syndrome, SBBYSS, artificial intelligence, deep learning

## Abstract

Tatton–Brown–Rahman syndrome (TBRS) and Say–Barber–Biesecker– Young–Simpson variant of Ohdo syndrome (SBBYSS) are extremely rare genetic disorders with less than 100 reported cases. Patients with these disorders exhibit a characteristic facial dysmorphism: TBRS is characterized by a round face, a straight and thick eyebrow, and prominent maxillary incisors, whereas SBBYSS is characterized by mask-like facies, blepharophimosis, and ptosis. The usefulness of Face2Gene as a tool for the identification of dysmorphology syndromes is discussed, because, in these patients, it suggested TBRS and SBBYSS within the top five candidate disorders. Face2Gene is useful for the diagnosis of extremely rare diseases in Korean patients, suggesting the possibility of expanding its clinical applications.

## 1 Introduction

An overgrowth–intellectual disability (OGID) syndrome with craniofacial dysmorphisms and autistic features, termed Tatton–Brown–Rahman syndrome (TBRS, OMIM #615879), was clinically reported together with the causative gene (*DNMT3A*) in 2014. TBRS is caused due to a heterozygous mutation in *DNMT3A* on chromosome 2p23 and is an extremely rare OGID syndrome, with approximately 80 cases reported till date (Balci et al., 2020). This syndrome is characterized by macrocephaly noticed at birth, joint hyperlaxity, scoliosis, hypotonia, and seizures, identical to the characteristics of Sotos syndrome, which is also an OGID syndrome (Manor and Lalani, 2020). However, the facial dysmorphism is different and includes a round face, straight and thick eyebrows, and prominent maxillary incisors. Sotos syndrome is the most common of the OGID disorders, with other syndromes in this category including Weaver syndrome and TBRS (Tatton-Brown et al., 2017; Manor and Lalani, 2020). Because it has mild dysmorphic features compared with other OGID syndromes, it is difficult to diagnose TBRS based on the facial phenotype alone.

Another extremely rare genetic disease, Say–Barber–Biesecker–Young–Simpson variant of Ohdo syndrome (SBBYSS, OMIM #603736), is one of the disorders caused by *KAT6B* mutation, that include genitopatellar syndrome (GPS). To date, 58 patients with SBBYSS, 18 patients with GPS, and 13 additional patients with intermediate phenotypes between these two syndromes have been reported to have *KAT6B*-related disorders (Lemire and Lee, 2012). The condition presents in infancy with severe hypotonia and feeding problems (Lonardo et al., 2019). The distinctive facial appearances of these patients include mask-like facies, blepharophimosis, and ptosis. Moreover, prominent cheeks, low-set and posteriorly rotated ears, downslanting palpebral fissures, a flat broad nasal bridge, a bulbous nose, a long philtrum, a thin upper lip, a thin lip vermilion, and micrognathia are common features of GPS and SBBYSS (Lemire G, 2012). Considering these varied detailed features, clinical diagnosis based solely on facial dysmorphism is often challenging.

An increasing number of rare genetic syndromes such as TBRS and SBBYSS present a challenge to clinical geneticists. Latorre-Pellicer et al. reported that computer-aided image analysis based on deep learning could support the diagnosis of Cornelia de Lange syndrome, a rare genetic disease (Latorre-Pellicer et al., 2020). The Face2Gene application, which is a novel framework based on DeepGestalt developed by FDNA Inc (Boston, MA, United States), is a next-generation phenotyping technology. DeepGestalt performs recognition of two-dimensional frontal facial images to detect facial landmarks and facial subregions, followed by the analysis of clinical features. Gurovich et al. proposed a new technology for powering Face2Gene, i.e., DeepGestalt, which achieves 91% top-10 accuracy (Gurovich et al., 2019). DeepGestalt yields higher first-rank scores in individuals with a genetic syndrome than in those without a diagnosis of a genetic syndrome (Pantel et al., 2020). Although most of the clinical information deposited on Face2Gene stems from patients of European descent, the performance of this system on other populations has been demonstrated in several recent studies (Mishima et al., 2019). However, there are few reports of the successful use of Face2Gene to target extremely rare genetic diseases. In this case report, we aimed to evaluate the performance of Face2Gene by analyzing two cases of successful diagnosis of an extremely rare genetic syndrome in Korean patients: TBRS and SBBYSS. This study and its publication were approved by the patients’ families and the Internal Review Board of the main institution involved in this study.

## 2 Case report

### 2.1 1 Patient A

#### 2.1.1 Clinical presentation

This male patient was examined in the genetics clinic at the age of 5 years because of overgrowth, speech disturbance, and intellectual disability. He was born vaginally at full term, weighing 3,900 g. His family history was unremarkable. Although he walked independently at 16–17 months of age, his motor developmental milestone was within normal limits. At the age of 5 years, his height was 123 cm (SD, +2.54 cm), his weight was 33 kg (SD, +2.88 kg), and his head circumference was 53 cm (SD, +1.00 cm). The observed dysmorphisms included heavy and low-set horizontal eyebrows, hypertelorism, narrow palpebral fissures, a round face, and a thin upper lip. Additional clinical features included generalized hypotonia, scoliosis, and skew feet.

#### 2.1.2 Deep-neural-network-driven facial recognition (Face2Gene) evaluation

**FIGURE 1:**
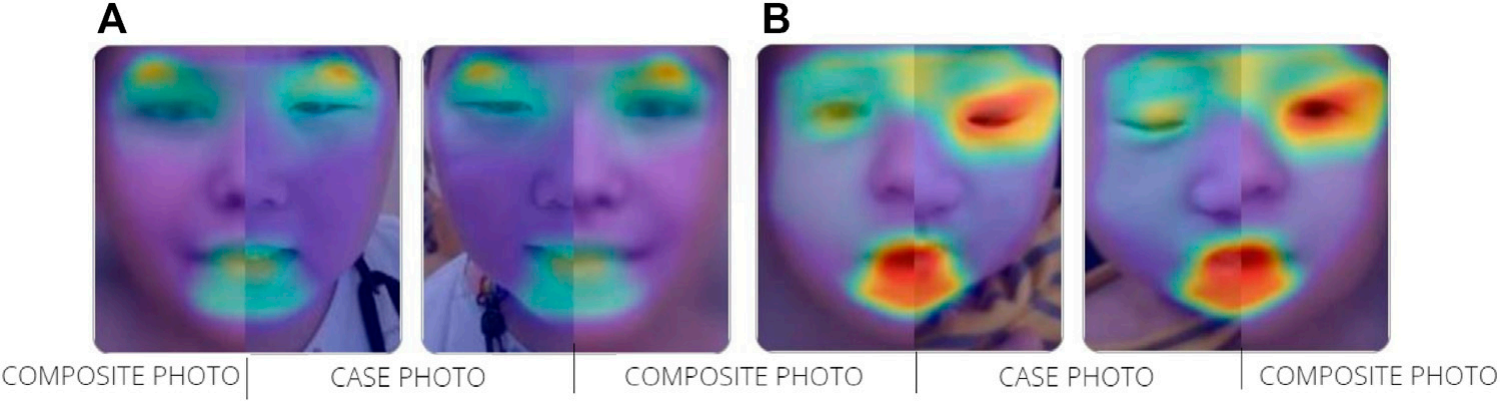
Overlapping facial regions of case photo and composite photo are indicated by the colored halo from red to blue **(A)** TBRS **(B)** SBBYSS.

**FIGURE 2:**
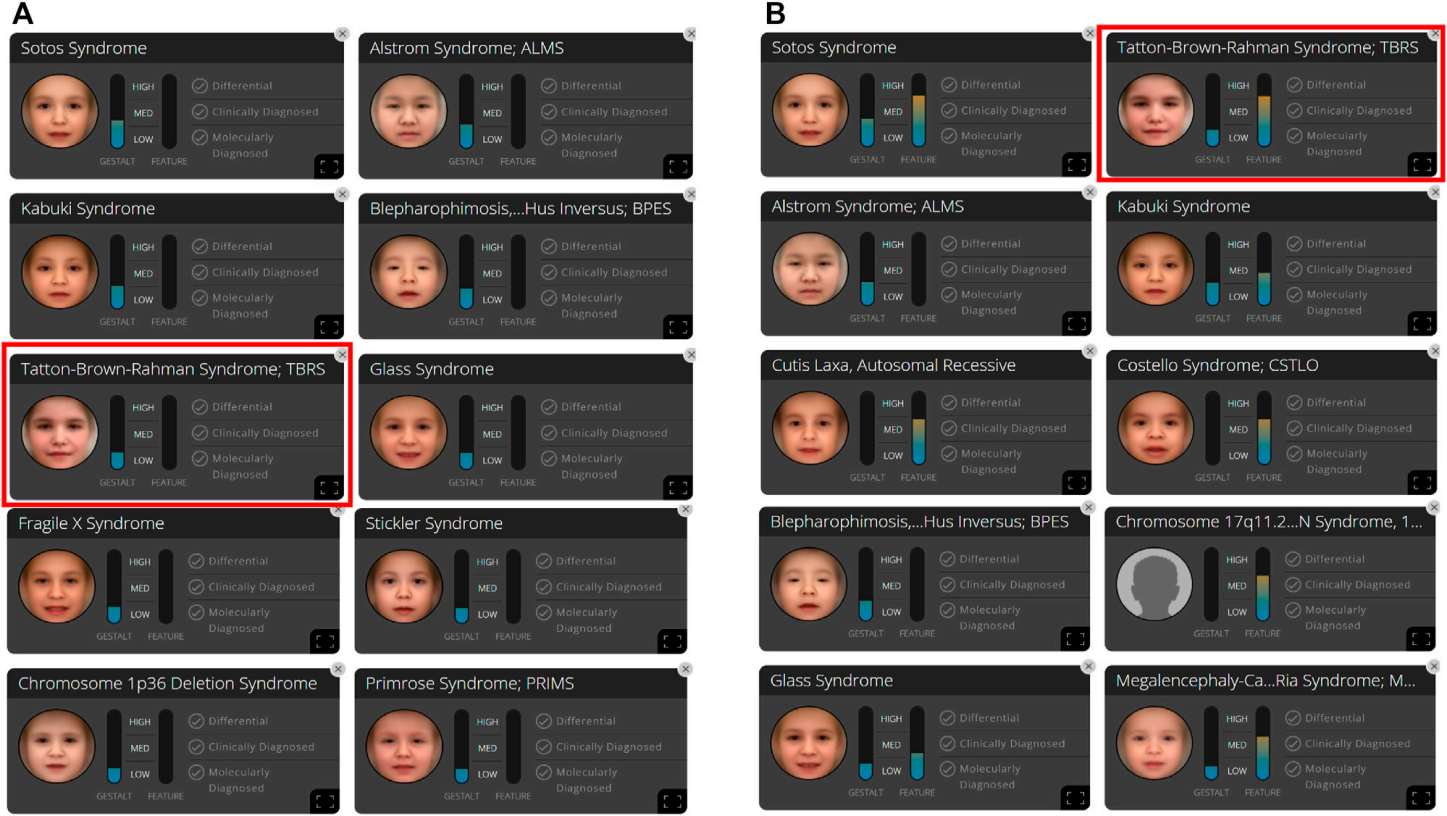
In patient A, 10 syndromes were automatically suggested by the Face2Gene platform as a tentative diagnosis based on **(A)** facial gestalt (frontal image) alone and **(B)** facial gestalt combined with clinical symptoms.

We analyzed the frontal image of the patient through the CLINIC application of the Face2Gene platform (FDNA Inc.; https://www.face2gene.com), which identified TBRS among the 10 automatically suggested syndromes as a tentative diagnosis based solely on facial gestalt. The typical features associated with TBRS included heavy and horizontal eyebrows and a thin upper lip, exactly as highlighted by the red color of the heat map, and other features such as hypertelorism, narrow palpebral fissure, and round face were highlighted by the green and blue colors of the heat map (Figure 1A). When analyzing only the frontal image, TBRS ranked fifth among the 10 suggested syndromes, and when the clinical features (generalized hypotonia, intellectual disability, macrocephaly, tall stature, scoliosis, and overgrowth) were included, TBRS ranked second (Figure 2).

#### 2.1.3 Genetic evaluation

At the time of the first visit of this patient to our institution, clinical exome sequencing was performed using a TruSight One expanded sequencing panel (Illumina Inc., San Diego, CA, United States), which identified a novel heterozygous nonsense variant, c.1279G>T (p.Glu427Ter), in the *DNMT3A* gene (NM_175,629.2). The variant was confirmed as being pathogenic according to the guidelines of the American College of Medical Genetics and Genomics (Richards et al., 2015) and was classified as a *de novo* mutation compared with the results obtained in the conventional Sanger sequencing of the parents.

#### 2.1.4 Other features

The neurodevelopmental evaluation of the patient showed intellectual disability with a Full-Scale IQ of 43 according to the Korean Wechsler Intelligence Scale for Children, fourth edition. Although a moderate sedation protocol was applied, brain magnetic resonance imaging (MRI) was not performed because of his lack of cooperation. He also had normal testing for fragile X syndrome, and biochemical screening for inborn errors of metabolism, including homocysteine levels, was also normal.

### 2.2 Patient B

#### 2.2.1 Clinical presentation

The patient was a 10-month-old boy and only child of healthy nonconsanguineous Korean parents. He was born at 38 weeks of gestation *via* cesarean section. He visited the developmental rehabilitation clinic with a general developmental delay. On physical examination, several dysmorphic features, including congenital blepharophimosis, epicanthus inversus, micrognathia, flat and broad nasal bridge, bulbous nasal tip, high-arched palate, small mouth, and overriding toes, were observed. His body weight was 8.7 kg (SD, −0.95 kg), his height was 73.4 cm (SD, +0.13 cm), and his head circumference was 43 cm (SD, −1.9 cm) at 10 months of age. The two testes were undescended, and bilateral orchiopexy was performed.

#### 2.2.1 Deep-neural-network-driven facial recognition (Face2Gene)

**FIGURE 3:**
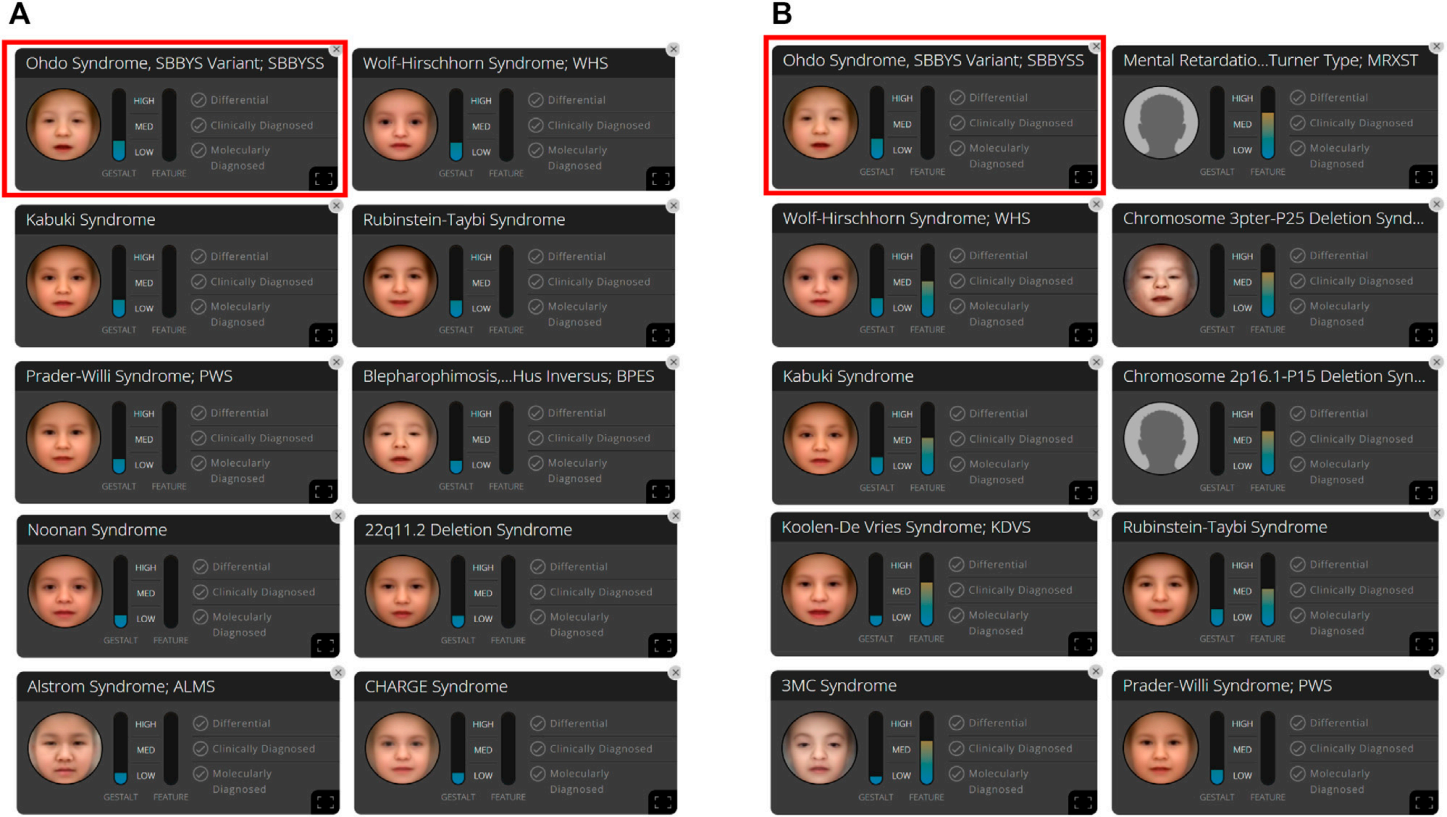
In patient B, 10 syndromes were automatically suggested by the Face2Gene platform as a tentative diagnosis based on **(A)** facial gestalt (frontal image) alone and **(B)** facial gestalt combined with clinical symptoms.

At the time of the first visit, the frontal image of the patient obtained through the CLINIC application of the Face2Gene platform was analyzed. The patient exhibited the typical features associated with SBBYSS, i.e., blepharophimosis, epicanthus inversus, micrognathia, thin upper lip, and thin lip vermilion, exactly as highlighted by the red color of the heat map, and other features such as a flat broad nasal bridge and bulbous nose were highlighted by the green and blue colors of the heat map (Figure 1B). In both frontal image and clinical feature analyses, SBBYSS ranked first among all diagnoses (Figure 3).

#### 2.2.3 Genetic evaluation

Clinical exome sequencing was performed using a TruSight One expanded sequencing panel (Illumina Inc., San Diego, CA, United States). A novel heterozygous frameshift deletion, c.4911_4921del p (Val1638AlafsTer27), in *KAT6B* was identified on the basis of the reference sequence NM_012,330.3 and was classified as a pathogenic variant according to the guidelines of the American College of Medical Genetics and Genomics (Richards et al., 2015). The patient’s parents were advised to undergo testing for the confirmation of a *de novo* mutation, but they refused this examination.

#### 2.2.4 Other features

A developmental assessment (Bayley Scales of Infant and Toddler Development) administered at the age of 10 months revealed an overall developmental age of 5–6 months. An echocardiogram showed an atrial septal defect secundum (8 mm) and a left-to-right shunt. Brain MRI showed infantile CSF space widening in bilateral frontotemporal regions.

## 3 Discussion

In the present study, we described the power of the DeepGestalt program to analyze facial dysmorphic features in patients with extremely rare genetic diseases. The diagnosis of rare diseases is one of the most challenging areas of clinical genetics. Prior to this study, we believed that the DeepGestalt program would only be successful in the identification of common genetic conditions; however, it has also succeeded in identifying patients with rare genetic disorders (Gardner et al., 2017; Liehr et al., 2018; Arora et al., 2020; Pascolini et al., 2020; Tripon et al., 2020), as in our case. For the diagnosis of rare diseases, the input of clinical findings as well as facial gestalt analysis are recommended (Elmas and Gogus, 2020).

When we analyzed the frontal image of patient A, the heat map compared to the composite photo of Sotos syndrome was obtained. It can be considered that downslanted palpebral fissures, hypertelorism, and prognathism with a pointed chin are strongly suggestive of Sotos syndrome before the appearance of clinical features (Manor and Lalani, 2020). In comparison with the composite photo of TBRS, the narrow palpebral fissures were highlighted in green color, whereas the downslanted palpebral fissures were highlighted in red in comparison with the composite photo of Sotos syndrome. It seems that eye characteristics acted as a factor for the detection of Sotos syndrome more strongly than TBRS, and this is thought to be based on data from Face2Gene application. The clinical features of patient A include generalized hypotonia, intellectual disability, macrocephaly, tall stature, scoliosis, and overgrowth. All these features were observed in Sotos syndrome; thus, even when clinical features are considered, the diagnosis of Sotos syndrome was more appropriate than TBRS.

TBRS has mild dysmorphic features compared with other OGID syndromes. Therefore, as in the analysis of the Sotos syndrome above, even if the patient has only one more powerful feature suggesting another syndrome, it may be difficult to make an accurate diagnosis based solely on facial gestalt. However, in Weaver syndrome, which is another OGID syndrome, hypertelorism (widely spaced eyes), large ears, and protruding chin are the main clinical features (Manor and Lalani, 2020). Of these, the only feature that was observed in this patient was hypertelorism, so Weaver syndrome was not suggested as a tentative diagnosis. Therefore, it can be considered that the probability of the diagnosis of syndromes not included in the upper ranks of 10 suggested syndromes is low. Upon combing the clinical features, the suggested ranking of the actual diagnosis could be higher, as in our case, and can be used as an excellent screening tool.

An ethnic bias in the training data sets that contain mostly Caucasian faces has been presented as an obstacle to this algorithm. In a study of children with an intellectual disability, Face2Gene showed a better recognition rate for Down syndrome in Caucasians (80%) compared with Africans (36.8%) (Lumaka et al., 2017). In contrast, studies of recent versions of DeepGestalt suggested that ethnicity had no major effect on its sensitivity (Vorravanpreecha et al., 2018; Mishima et al., 2019). It has been reported that Face2Gene was useful when matched by analyzing only the face gestalt in Korean patients who had already been molecularly diagnosed (Jang, 2017). Compared with the Caucasian face, the Asian face is characterized by a greater intercanthal width, epicanthal folds, a smaller eye fissure length, a smaller oral width, a greater mandibular width, chin retrusion, and a nose with a wider base and less tip projection (Le et al., 2002; Farkas et al., 2005; Gu et al., 2011; Liew et al., 2020). If these characteristics are considered during face gestalt analysis, the diagnostic accuracy of Face2Gene will increase.

Kruszka et al. presented that face analysis technology using Face2Gene in a diverse population including Asians can distinguish the general population from the genetic diseases (Down syndrome, Turner syndrome, Noonan syndrome and 22q11.2 deletion syndrome) (Kruszka et al., 2017a; Kruszka et al., 2017b; Kruszka et al., 2017c; Kruszka et al., 2020). The authors analyzed that several consistent clinical findings that were found to be independent of ethnicity may have supported the accurate diagnosis of these genetic disorders. In a recent study, Li et al. detected P/LP (pathogenic/likely pathogenic) variants related to 52 genes in 131 (70.4%) of 186 cohorts with short stature with facial dysmorphism confirmed (Li et al., 2022). As presented in above studies, it has been suggested in large sample studies that Face2Gene can be useful in diagnosing genetic diseases with facial dysmorphism in Asians as well. However, a more comprehensive analysis with a larger sample size is needed to demonstrate the usefulness of Face2Gene in diagnosing extremely rare diseases in Asians.

The assessment of facial dysmorphism is subjective, can be time-consuming, and requires a high level of clinical expertise. Face2Gene can quickly identify suspected diseases and reduce costs by conducting targeted gene tests. Recent studies have demonstrated Face2Gene is as good as or superior to clinical assessments performed by trained healthcare providers in identifying genetic disorders (Basel-Vanagaite et al., 2016; Gripp et al., 2016; Liehr et al., 2018; Pantel et al., 2018; Vorravanpreecha et al., 2018). In a very recent study, GestaltMatcher, an encoder based on deep convolutional neural networks, matched patients with other patients with the same molecular diagnosis, even if the disorder was not included in the training set (Hsieh et al., 2022). This program could accelerate the clinical diagnosis of patients with extremely rare diseases. To build an artificial intelligence solution within genetics, the integrity of the data, ethical and privacy policies, and trust in the workflow should be established (Gurovich, 2020).

In conclusion, we report the usefulness of Face2Gene as a tool for the identification of dysmorphic syndromes that suggested TBRS and SBBYSS within the top five candidate disorders in these patients, suggesting the possibility of expanding its clinical applications. In order to prove the usefulness of Face2Gene in diagnosing extremely rare diseases in Korean patients, a more comprehensive analysis including various extremely rare genetic diseases with a larger sample size is needed. It is expected that additional cases will accumulate in the future, to enable a detailed diagnosis using Face2Gene.

## Data Availability

The datasets for this article are not publicly available due to concerns regarding participant/patient anonymity. Requests to access the datasets should be directed to the corresponding author.
